# Metformin and Exercise in Cancer: Better Together

**DOI:** 10.1093/jncics/pkz097

**Published:** 2019-11-20

**Authors:** Vuk Stambolic, Ryan J O Dowling

**Affiliations:** 1 Princess Margaret Cancer Centre, University Health Network, Toronto, ON, Canada; 2 Department of Medical Biophysics, University of Toronto, Toronto, ON, Canada

The levels of obesity in the general population are reaching epidemic proportions. Over the last 40 years, the prevalence of obesity in the United States has tripled, and the World Health Organization estimates that 650 million individuals worldwide older than 18 years are obese. Although it has long been recognized that obesity is a key risk factor in the etiology of type 2 diabetes and cardiovascular disease, emerging evidence indicates that obesity is an adverse factor in the development and severity of many human cancers, including those of the breast and colon (1,2). Obesity is associated with changes in the levels of a variety of systemic physiological factors that may contribute to tumorigenesis and reduced patient outcomes, including adipokines, sex hormones, inflammatory cytokines, glucose, and insulin. There is great interest in modulating these factors through medications or lifestyle interventions for the prevention and/or treatment of cancer.

In their article published in this issue of the Journal, Meyerhardt and colleagues (3) report the results of a phase II trial conducted in colorectal and breast cancer patients randomly assigned to structured exercise, the antidiabetic drug metformin, a combination of these interventions, or neither for 12 weeks following completion of standard therapy. The exercise intervention consisted of in-person aerobic sessions twice a week and additional at-home aerobic activity, with the goal of reaching 220 minutes of moderate intensity exercise every week. Metformin was administered as 850-mg tablets taken twice daily. The primary monitored outcome was change in fasting insulin levels, with secondary analyses focused on metabolic and anthropometric markers. Metformin is a frequently prescribed insulin-lowering diabetic medication that exhibits anticancer effects in preclinical models and correlates with lower cancer incidence, severity, and adverse outcomes in human type 2 diabetes patients (4,5), and the impact of exercise on insulin and other metabolic markers is well documented. Ninety-one patients completed the study, with beneficial effects on the levels of insulin and metabolic factors observed in all three intervention arms. Fasting insulin levels statistically significantly decreased in response to all three treatments, with the combination of metformin and exercise producing the largest reduction (−2.47 μU/mL vs +2.79 μU/mL in the control arm). Similar effects of each treatment on body weight between baseline and 12 weeks were also found (−1.8% combination vs +1.55% in the control arm), and the combination treatment also led to considerable reductions in the homeostatic model assessment (HOMA, a measure of insulin resistance) and the adipokine leptin compared with the control arm. These results clearly demonstrate beneficial effects of exercise and metformin on insulin, body weight, and other factors known to promote cancer development and progression, with the combination suggestive of synergy, although the small sample size precluded formal analysis. Numerous lines of evidence support the notion that insulin mediates some of the adverse effects of obesity in a variety of cancers. Increased insulin levels are associated with breast cancer recurrence and death, and elevated fasting insulin levels and c-peptide (cleaved from proinsulin) have been linked to the recurrence of colorectal adenomas and mortality in patients with colorectal cancer (6–9). Furthermore, elevated body weight is associated with risk of at least 13 different cancers and higher mortality in breast and colorectal cancer patients (10–12).

The use of lifestyle interventions and medications that modulate metabolic factors has been considered for the prevention and treatment of cancer for years, with the recent drastic increase in the incidence of obesity only heightening the interest in these types of interventions. Multiple studies have evaluated various exercise and weight loss regimens and their impact on metabolic physiology in patients with cancer, but few have yielded robust data on cancer outcomes. Recent data from the Women’s Health Initiative Observational Study indicate that intentional weight loss (5% of total body weight) was associated with reduced risk of obesity-related cancers, particularly endometrial cancer (13). Likewise, use of metformin as an anticancer agent has been explored in a number of preclinical and clinical studies. Metformin can reduce insulin, glucose, leptin, and other key metabolic factors implicated in tumorigenesis in nondiabetic patients with cancer, but its impact on patient outcome in prospectively designed trials has not been entirely positive (14,15) despite some recent encouraging results (16).

The current study by Meyerhardt and colleagues is a rare example of a trial that has examined the combination of metformin with an exercise intervention in the context of cancer. Although limited by the small sample size and relatively low compliance to metformin, the results conclusively demonstrate that the combination was most effective at reducing insulin, body weight, HOMA, and leptin in breast and colorectal cancer survivors, highlighting the potential utility of metformin and exercise in patient management and care in oncology. Of particular interest, in exploratory analyses there were greater effects of metformin, exercise, and especially the combination of the two on insulin, glucose, HOMA, and body mass index (BMI) in patients with higher baseline values for each of these variables. This implies that patients with the highest BMI, or those with the most severe metabolic dysfunction, may experience the greatest benefit of metformin and exercise and raises the possibility that stratification strategies based on those parameters may become instrumental in predicting response in future cancer trials of metabolic medications, weight loss, or exercise regimens. These findings emphasize the importance of incorporating standard patient characteristics, such as body weight, BMI, and blood insulin, in the design and execution of future clinical trials.

It is estimated that by the year 2030, 50% of Americans will be considered formally obese (17). Given the strong association between obesity and cancer risk and outcome, novel treatments are required to combat the obesity-associated metabolic disruptions that drive cancer development and progression. The trial by Meyerhardt and colleagues provides strong evidence that medications and lifestyle interventions are effective options for improving the physiology of patients with cancer, with possible beneficial effects on patient outcome. Large prospectively designed clinical trials are required to build on these important findings and evaluate the efficacy of such interventions in the context of cancer prevention and recurrence. Indeed, large phase III trials such as Canadian Cancer Trials Group MA.32 (CCTG MA.32) (18) and Breast Cancer Weight Loss (BWEL) (19) are well positioned to evaluate the effects of metformin and weight-loss programs on patient outcomes as well as to provide extremely valuable insight into possible patient stratification strategies. Treatments that effectively reduce the adverse effects of obesity on cancer have the potential to considerably improve disease management and patient survival, which would alleviate much of the rising economic burden of obesity on health-care costs. Indeed, the use of metformin, a generic, off-patent drug, in combination with exercise regimens represents an inexpensive treatment option for cancer patients, with broadly beneficial implications worldwide.

## Notes

Affiliations of authors: Princess Margaret Cancer Centre, University Health Network, Toronto, ON, Canada (VS, RJOD); Department of Medical Biophysics, University of Toronto, Toronto, ON, Canada (VS, RJOD).

The authors have no conflicts of interest to disclose.
